# The Effect of Using Mobile Technology-Based Methods That Record Food or Nutrient Intake on Diabetes Control and Nutrition Outcomes: A Systematic Review

**DOI:** 10.3390/nu8120815

**Published:** 2016-12-17

**Authors:** Judi Porter, Catherine E. Huggins, Helen Truby, Jorja Collins

**Affiliations:** 1 Department of Nutrition and Dietetics, Monash University, Notting Hill 3168, Australia; Catherine.huggins@monash.edu (C.E.H.); helen.truby@monash.edu (H.T.); jorja.collins@monash.edu (J.C.); 2 Allied Health Research Office, Eastern Health, Box Hill 3128, Australia

**Keywords:** mobile electronic devices, mobile applications, diabetes, nutrition outcomes

## Abstract

(1) Background: Mobile technologies may be utilised for dietary intake assessment for people with diabetes. The published literature was systematically reviewed to determine the effect of using mobile electronic devices to record food or nutrient intake on diabetes control and nutrition outcomes; (2) Methods: The review protocol was registered with PROSPERO: registration number CRD42016050079, and followed PRISMA guidelines. Original research of mobile electronic devices where food or nutrient intake was recorded in people with diabetes with any treatment regimen, and where this intervention was compared with usual care or alternative treatment models, was considered. Quality was assessed using the Quality Criteria Checklist for Primary Research; (3) Results: Nine papers formed the final library with a range of interventions and control practices investigated. The food/nutrient intake recording component of the intervention and patient engagement with the technology was not well described. When assessed for quality, three studies rated positive, five were neutral and one negative. There was significantly greater improvement in HbA1c in the intervention group compared to the control group in four of the nine studies; (4) Conclusion: Based on the available evidence there are no clear recommendations for using technology to record dietary data in this population.

## 1. Introduction

The use of mobile technology in everyday life continues to increase exponentially. By 2019, the number of smartphone users worldwide is expected to grow to 2.7 billion, while there will be 1.4 billion tablet users [1,2]. Rates of diabetes in China (11.6%) [3] have now overtaken those reported in the United States (9.3%) [4] with diabetes considered to be one of the most challenging health problems of the 21st century [5]. There are vast opportunities for interventions for diabetes that utilise or are delivered through mobile phones or tablets (i.e., mobile applications) or other portable devices.

Recommendations for optimal management of diabetes have been outlined by the American Diabetes Association [6]. There should be organised and coordinated approaches with collaboration between the health care team and the patient who has an active role in their self-management. Measuring, monitoring, analysing, communicating and acting on a range of parameters, in addition to blood glucose levels, are integral to effective management. Mobile technologies provide an interface for this to occur, and there is some evidence of their effectiveness. For example, a systematic review identified statistically significant and clinically relevant declines in HbA1c levels for adults receiving telemedicine applications with personalised feedback compared to non-telemedicine treatment approaches [7]. Technology has also been shown to be preferential to weighed food records for recording dietary intake information in people with type 2 diabetes [8].

A recent systematic review [9] evaluated 65 free to download English language applications for the self-management of diabetes. Whilst there was a large number (956 applications across Google Play, App Store and Windows Phone Store) and wide selection of applications available, reviewers identified that the versatility and inclusion of features was highly variable. Only eight of the applications reviewed included a food database with energy, carbohydrate or fluid intake input, with an average time for meal log of entry of less than one minute to five minutes [9]. 

The effect of mobile devices where food or nutrition intake is recorded has been considered in other chronic conditions including weight management [10] and chronic renal disease [11]. These reviews reported varying levels of effect, whilst opportunities for further development of applications were identified. No similar review has been undertaken of studies comparing the use of mobile devices for recording nutritional intake compared with usual care in diabetes management. We aimed to systematically review the published literature to determine the effect of using mobile electronic devices to record food or nutrient intake on diabetes control and nutrition outcomes.

## 2. Methods

The review protocol was registered prospectively with PROSPERO, registration number CRD42016050079. In a deviation to the registered protocol, interventions were extended from mobile applications only (mobile phone or tablet) to include mobile electronic devices (including stand-alone portable devices such as personal digital assistants (PDA)). The PRISMA statement [12] was followed throughout all stages of the review.

### 2.1. Eligibility Criteria

The PICO (Participant–Intervention–Comparator–Outcomes design) format of Shamseer et al. [13] was used to develop criteria for review inclusion. Original research among people with type 1 or 2 diabetes mellitus or gestational diabetes (excluding pre-diabetes or diabetes prevention) with any treatment regimen, using mobile electronic devices where food or nutrient intake was recorded (alone or in addition to other parameters) and compared with usual care or alternative treatment models was considered. No age or gender restrictions were applied. Interventions consisting of text messages, phone calls, and access to internet or websites only were ineligible, although these were acceptable if delivered in addition to the intervention of interest described above. Studies with no intervention (e.g., cross-sectional studies) or no control group (e.g., before-and-after studies), reviews, opinions or commentaries, protocol papers, conference abstracts, book chapters and case reports were excluded from the review.

HbA1c was the primary outcome due to its value in assessing treatment effectiveness in patients with diabetes. It reflects glycemic control over a 3 months or 90 days average plasma glucose concentration whilst being correlated with microvascular and neuropathic complications [6]. Secondary outcomes were: other nutritional outcomes (blood glucose levels, anthropometric measures and lipids), participant engagement and adherence with nutritional data entry, and satisfaction and feasibility of nutritional data entry.

### 2.2. Search Terms

**Figure 1:**
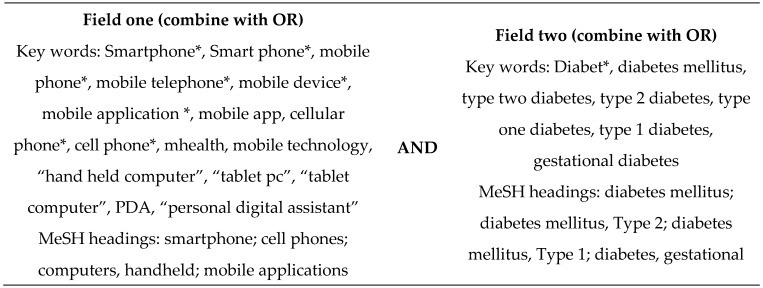
Search strategy used in the systematic literature review of the effect of using mobile devices to record food or nutrient intake on diabetes control and nutrition outcomes. * used to retrieve unlimited suffix variations.

Search terms with a focus on the population and intervention were determined through the examination of key words used in the relevant literature. The search strategy used is shown in Figure 1. No limits were applied. 

### 2.3. Study Selection

Six databases, Ovid MEDLINE, Embase, CINAHL, EBM Reviews-Cochrane Database of Systematic Reviews, PsycINFO and EBM Reviews-Health Technology Assessment, were searched to identify publications of relevance from date of commencement to September 2016. Reference lists of papers included in the final library were also reviewed to identify additional studies for inclusion. 

The process of identification, screening and eligibility assessment was applied to ensure that all relevant studies were included. After duplicates were removed, two authors independently screened titles and abstracts, then full text publications (J.P., J.C.). Where conflicting opinions arose, these were resolved through consensus.

### 2.4. Data Extraction and Quality Assessment

A template was developed to extract relevant data from the original papers with data extraction completed by one author (J.C.). Two authors independently rated study quality using the Quality Criteria Checklist for Primary Research (J.P., C.E.H.) [14]. This tool considers aspects of dietary measurement and error, and is specific for studies in nutrition and dietetics. A rating of negative (weak quality, generalisability, data collection and analysis, likely bias), neutral (neither exceptionally strong nor exceptionally weak quality) or positive (strong quality, generalisability, data collection and analysis, limited bias) was assigned for each study using the rating scale provided by the checklist. Where details were not provided or authors considered an “unsure” response for specific aspects within each validity question, a final response of “no” was made.

### 2.5. Synthesis of Results

Results were described narratively. Due to the large variance in the types and length of interventions (including the input of nutrition data), authors considered that a meta-analysis was inappropriate.

## 3. Results

**Figure 2:**
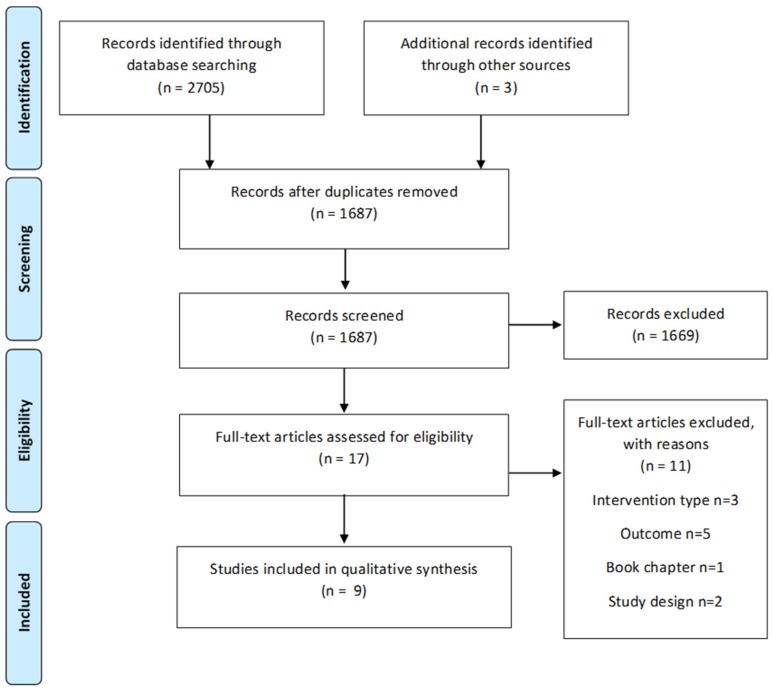
Study selection process.

The study selection process is illustrated in Figure 2. The database search yielded 2705 records. The titles and abstracts were screened for 1687 records, with full text reviewed for 17 manuscripts. Of these, six studies were included [15,16,17,18,19,20] in addition to one study [21] known to authors and two studies [22,23] identified from hand searching of reference lists, with a final library of nine studies [15,16,17,18,19,20,21,22,23]. No previous reviews of the research question were identified.

Participants were adults with type 1 diabetes [15,16,22], type 2 diabetes [17,18,19], or either condition [20]. Tsang et al. [23] did not report the participant group. No studies undertaken in women with gestational diabetes were identified. Studies were identified from Europe [15,16,21,22], the United States [17,18] and Asia [19,20,23]. Study characteristics are described in Table 1.

In all included studies the intervention was a multi-component diabetes management strategy, where dietary data was recorded in addition to a range of other medical information (e.g., blood glucose levels, medications, physical activity). In some studies, only carbohydrate intake [15,16,18,20,22] was recorded and in others food/meal intake [17,19,21,23] was collected. Interventions were delivered via a mobile phone applications for three studies [15,20,21] and as a ‘system’ or ‘software’ accessible via mobile phone for four studies [16,18,19,22]. One study utilised a PDA [17] and another [23] a purpose-developed hand held device. Some studies included additional components as part of the intervention. 

Eight of the studies were randomised controlled trials (RCT) [15,16,17,18,19,20,21,22] whilst one [23] was of cross-over design. In some interventions the dietary input was analysed by a database or a clinician and made available to the patient [19,23] but this was unclear or not reported in other studies. Overall, the nutrition component of the application was poorly described in most studies with a lack of information on what information was captured, whether items were selected from a database of foods and if nutrient analysis was provided to the patient. 

The assessment of quality across the included library yielded variable results (Table 2). Three studies rated positive [16,17,21], five neutral [15,18,19,20,22] whilst one negative [23] due to a range of methodological and reporting concerns. Two particular issues were consistently noted. Bias was introduced through the participant selection process in many studies whereby only those who owned or had internet access or could reportedly use the technological device were eligible for inclusion. Although this may have enhanced study completion rates, the opportunity to participate was effectively only offered to a subsample of those with diabetes. This type of selection bias may make the intervention more relevant to the population being studied as participants were already engaged in mobile technology; this cannot be determined without understanding the reasons why some people with diabetes are not engaged in the use of technology. The statistical analysis in several cases were not reproducible, particularly the intention to treat analysis of RCTs. 

There was a statistically significantly greater improvement in HbA1c in the intervention group compared to the control group in four of nine studies [18,19,20,23]. Due to the multiple and varied components of the intervention and usual care, it was not possible to attribute whether the effect (or lack of) on HbA1c was attributable to recording of food or nutrient intake using a mobile device.

Secondary outcome data are reported in Table A1. Two studies [19,20] reported significant improvements in fasting blood glucose in favour of the intervention, alongside reductions in HbA1c. There were no significant improvements in other nutritional outcomes associated with the intervention. One study [22] reported a significantly greater reduction in triglycerides (but not other blood lipids) among patients with type 1 diabetes using mobile software with capacity to record blood glucose and carbohydrate. Patients’ engagement in the food and nutrient recording aspect of the intervention was not reported in all but two studies. Waki et al. [19] reported that 40% of participants recorded dietary intake data, with a drop off observed over time. In the study by Tsang et al. [23], the frequency of dietary data transmission by participants to obtain nutrient analysis was highly variable, while the majority (73%) of participants transmitted data for three meals at once. Where assessed [15,19,20,23], patients were satisfied with the intervention, but this did not specifically relate to the food or nutrient recording component. 

## 4. Discussion

Given the available number of diabetes applications, the size of the included library evaluating the effect of using mobile electronic devices to record food or nutrient intake on diabetes control and nutrition outcomes was smaller than anticipated. Several studies rated strongly from a quality perspective, although bias was introduced through participant selection and lack of methodological description. 

Some studies did report favourable effects of the intervention on HbA1c however these were not in the majority. All interventions captured multiple pieces of information (e.g., blood glucose, physical activity) in addition to dietary data. Some interventions also included other components such as interaction with health professionals. Consequently, in the studies where benefits were seen, it was not possible to attribute this to the recording of dietary information alone. Mobile applications that record food and nutrient intake data only (e.g., Easy Diet Diary) are available, but their effect is unknown as they were not utilised in the studies included in this review. It is notable that in all four studies where there were significant benefits for HbA1c, the intervention involved providing the patient with analysis and/or feedback from a clinician on data captured. It is possible that recording of diabetes related information alone is insufficient to affect outcomes with benefits instead arising from multifactorial diabetes management. 

Furthermore, specific detail relating to the nutritional component of the interventions, or the engagement and update by participants was not well described. This was not anticipated, but may be explained by the fact that most interventions were multi-component and did not intend to evaluate this aspect specifically. Evidently for there to be an effect, patients must comply with recording of dietary data, and reporting on adherence is essential. In most studies, inclusion criteria were having or being familiar with a mobile phone. While this excluded a proportion of patients with diabetes, it means that participants recruited were an appropriate target group for a technological based intervention. 

Subsequently, we are unable to make draw conclusions as to whether using mobile devices to record food or nutrient intake affects clinical outcomes, or give recommendations as for the development or enhancement of the nutritional models used within diabetes applications. It appears that there is no harm in using mobile devices to record dietary information among patients who are familiar with mobile phone technology. 

The results of this review contrasts with other reviews of the effectiveness of nutrition focused mobile applications on clinical outcomes in chronic disease populations. In overweight and obese populations with cardiovascular risk factors, Stephens et al. [10] identified beneficial impacts of text messaging or smart phone applications for reducing physical inactivity and/or overweight/obesity. A review in patients with chronic renal failure [11] found potential for clinical benefits, but no significant changes in nutritional outcomes (energy, protein, potassium, phosphorus or fluid intake, and intradialytic weight gain) through interventions of mobile application.

Some limitations at the study and review level were noted. The choice of HbA1c as a primary outcome may have limitations in several clinical conditions that affect HbA1c readings (e.g., certain anemias and in situations of abnormal red cell turnover) [6]. Consistent with the systematic review of telemedicine in the diabetes population [7], blinded outcome assessment was not commonly implemented. However the use of objective biochemical measures provides low risk of bias in their assessment. Conversely, strengths to the review include that there were no date or language restrictions applied, and the search strategy was wide reaching through the use of broad search terms and multiple databases.

There are opportunities for clinicians to engage with application developers to develop models for clinical trials. Maintaining the currency of the mobile devices (and applications) presents a challenge to program developers. Two studies in this review utilised PDAs/purpose developed handheld devices which have been now been superseded by smartphones and tablets. Carrying a second device for diabetes monitoring in addition to a mobile phone would likely be considered cumbersome. Future studies should report specific details relating to uptake and engagement of the participant with the intervention, the application (including process of nutrition data entry and use of a database) and the involvement and role of clinicians to enable reproducibility and comparison with other applications and studies. The use of HbA1c as a primary outcome would enable comparison between other studies that were limited to reporting of glucose monitoring in the absence of nutrition intake assessment. In order to fully understand the impact on diabetes of self-monitoring of diet using mobile devices, large RCTs are required comparing this intervention with a control group where dietary information is recorded using traditional methods (i.e., paper based diary) or not recorded at all.

## 5. Conclusions

Although technology may offer novel solutions to support measurement of dietary intake and improve clinical outcomes in people with diabetes, based on the present evidence, we are unable to define clear recommendations for nutrition technology use in this population.

## Figures and Tables

**Table 1:** Key characteristics and primary outcome data of studies comparing the effect of using mobile devices to record food or nutrition intake to usual care on diabetes control and nutrition outcomes.

**Author**	Drion et al., 2015 [15]	Forjouh et al., 2014 [17]	Quinn et al., 2011 [18]	Rossi et al., 2013 [16]	Waki et al., 2014 [19]	Zhou et al., 2016 [20]	Tsang 2001 [23]	Rossi et al., 2010 [22]	Holman et al., 2014 [21]
**Study Design**	RCT	RCT (4 arms)	Cluster RCT (4 arms)	RCT	RCT	RCT	Cross over study	RCT	RCT (3 arms)
**Duration**	3 months	12 months	12 months	6 months	3 months	3 months	3 months (each group)	6 months	1 year
**Setting**	Netherlands, 1 outpatient clinic	United States, 7 outpatient clinics	United States, 26 primary care practices	Italy, 12 outpatient clinics	Japan, 1 hospital	China, 1 hospital endocrinology department	Hong Kong, 1 outpatient clinic	Multinational. 3 outpatient clinics in Italy, 2 in England and 2 in Spain	Norway, 2 study centres, local clinics, diabetes courses and advertisements
**Population and Characteristics Mean (SD)**	Adults with T1DM. 33 (21) years, 63% male, BMI 26 (4) kg/m^2^, baseline HbA1c 62 (16) mmol/mol.	Adults with T2DM and HbA1c ≥7.5%. 58 (11) years, 45% male, BMI 34 (7) kg/m^2^, baseline HbA1c 9.3 (1.6) mmol/mol.	Adults (18–64 years) with T2DM and HbA1c ≥7.5%. 53 years, 49.7% male, 76% obese, baseline HbA1c 9.4 mmol/mol.	Adults with T1DM and HbA1c ≥7.5%. 34 (10) to 38 (10) years, 46 to 19% male, BMI 24 (4) to 25 (4) kg/m^2^, baseline HbA1c not reported.	Adults with T2DM. 57 (10) years, 66% male, 50% BMI <25, baseline HbA1c 7.1 (0.9)%	Adults with T1DM or T2DM. 53.5 (12.4) to 55.0 (13.1) years, 54%–60% male, BMI 23 (4) kg/m^2^, baseline HbA1c 9.8 (2.5) to 9.9 (2.4)%	Not reported. 30 (8) to 35 (8) years, 63% male, BMI 22 (3) to 26 (6) kg/m^2^, baseline HbA1c 8.5 (1.8) to 8.8 (1.8)%	Adults with T1DM. 35 (9) to 36 (9) years, 41%–44% male, baseline HbA1c 8.2 (0.8) to 8.4 (0.7)%	Adults with T2DM and HbA1c ≥7.1%. 57 (12) years, 59% male, BMI 32.7 (6.1) kg/m^2^, baseline HbA1c 8.2 (1.1)%
**Sample Size (*n*) Completion Rate**	63 (98%)	376 (70%)	213 (76%)	127 (88%)	54 (100%)	100 (100%)	20 (95%)	130 (92%)	151 (79%)
**Intervention/s Description**	Diabetes Under Control (DBEES) mobile app linked to a personal web portal. Captured BGL, medication, PA and CHO intake.	Intervention 1 (PDA) —Diabetes Pilot™ on a PDA. Captured BGL, BP, medication, PA and dietary intake using a food database. Intervention 2 (PDA + CDSMP)–As above plus Chronic Disease Self Management Program 6 week group education program to increase self efficacy.	Intervention 1 (coach) —Patient coaching and clinician support system on mobile phone and web. Captured BGL, CHO intake, medication.	Diabetes Interactive Diary (DID) software on mobile phone. CHO/insulin bolus calculator. Captured BGL and CHO intake, recorded using a “food atlas”.	DialBetics with FoodLog on mobile phone. Captured BGL, BP, pedometer readings and food intake recorded with photos, voice and text messages and linked to a database.	Welltang mobile app. Captured BGL, CHO intake, medications, notes.	Diabetes monitoring system (DMS) on hand held device. Captured BGL and food intake using a food database.	Diabetes Interactive Diary (DID) software on mobile phone. CHO/insulin bolus calculator. Captured BGL and CHO intake using a list of foods with pictures and quantities to select from.	Intervention 1 Few Touch Application (FTA) mobile app. Captured BGL, food intake, PA, goal setting and other information.
			Intervention 2 (CPP) coach + primary care provider portal—As above plus clinicians had access to data.						
									Intervention 2 Few Touch Application plus health counselling (FTA HC)—As above, plus 5 phone based sessions with diabetes nurse educator to improve self management.
			Intervention 3 (CPDS) coach + primary care provider portal + decision support—Coach program as above plus clinicians had access to analysed data linked to standards.						
**Communication between Patients and Clinician**	Not reported	Not reported	Yes, as above	Yes	Yes	Yes	Yes	Yes	Not reported
**Analysis of Food or Nutrient Data**	Not reported	Not reported	Yes, as above	Not reported	Yes	Not reported	Yes	Yes	Unclear
**Control/s Description**	Not reported	Control 1—Usual care; Control 2—CDSMP only (as described above)	Usual care	Usual care—standard education	Control group—unclear	Usual care—monthly clinic visits	Usual care—consultations with clinicians	Usual care—standard education	Usual care
**HbA1c**	No significant difference in change between groups (*p* not reported). Median (IQR) change. Control: 1 (−4–6) mmol/mol; Intervention: 1 (−1–2) mmol/mol.	No significant treatment effect (*p =* 0.771). Change Control: −0.7%; CDSMP: −1.1%; PDA: −0.7%, CDSMP + PDA: −1.1%	Significantly greater reduction in CPDS group compared to control (*p =* 0.001) and coach group compared to control (*p =* 0.027). No significant difference in change between CPP group and control (*p =* 0.40). Mean (95% CI) change coach: −1.6 (−2.3–−1.0)% CPP: −1.2 (−1.8–−0.5)% CPDS: −1.9 (−2.3–−1.5)% control: −0.7 (−1.1–−0.3)%	No significant difference in change between groups (*p =* 0.73). mean (SD) change DID group: 0.49 (0.11)%; Control: 0.48 (0.11)%	Significant difference in change between groups (*p =* 0.015). Mean change DialBeltics group: −0.4%; Control: 0.1%	Significantly greater reduction in the intervention group (*p <* 0.001). Mean change Welltang group: −1.95%; Control: −0.79%	Significant reduction associated with the intervention (*p <* 0.019). mean (95% CI) difference 0.825% (0.155–1.50).	No difference in change between groups (*p =* 0.68). mean change (SD). DID group: −0.4 (0.9)%; Control: −0.5 (1)%	No difference in change between groups (*p* not reported). Mean (95% CI) change. FTA: −0.31 (−0.67–0.05)% FTA HC: −0.15 (−0.58–0.29)%; Control: −0.16 (−0.5–0.18)%

RCT, randomised controlled trial; T2DM, type 2 diabetes; T1DM, type 1 diabetes; BMI, body mass index; BGL, blood glucose level; PA, physical activity; CHO, carbohydrate; BP, blood pressure; SD, standard deviation; app, application; HbA1c, Hemoglobin A1cDBEES, Diabetes Under Control; PDA, personal digital assistant; CDSMP, Chronic Disease Self Management Program; CPDS, coach + primary care provider portal + decision support; CPP, coach + primary care provider portal; DID, Diabetes Interactive Diary; DMS, Diabetes monitoring system; FTA, Few Touch Application; HC, health counselling.

**Table 2:** Quality assessment of studies comparing the effect of using mobile devices to record food or nutrition intake to usual care on diabetes control and nutrition outcomes.

Author/Year	Quality Rating ^a^	Validity Items ^b^										Example of Reasons for Downgrading
		1	2	3	4	5	6	7	8	9	10	
Drion et al., 2015 [15]	Neutral	√	x	√	√	x	x	√	√	√	√	Devices not provided hence biasing sample; methods were unclear
Forjouh et al., 2014 [17]	Positive	√	√	√	√	x	√	√	x	√	√	Methods used in the intention to treat analysis not described
Holman et al., 2014 [21]	Positive	√	√	√	√	x	√	√	x	√	√	Methods used in the intention to treat analysis not described
Quinn et al., 2011 [18]	Neutral	√	x	√	√	x	√	√	√	√	√	All participants needed internet and email access.
Rossi et al., 2010 [22]	Neutral	√	x	√	√	x	√	√	√	√	x	Participants were required to be familiar with mobile phones, and be in possession of, a mobile phone card.
Rossi et al., 2013 [16]	Positive	√	√	√	√	x	√	√	x	√	√	Methods used in the intention to treat analysis not described
Tsang et al., 2001 [23]	Negative	x	x	x	√	x	x	x	x	√	√	Selection of study groups and statistical analysis not clearly reported.
Waki et al., 2014 [19]	Neutral	√	x	x	√	x	x	√	x	√	x	Methods used in the intention to treat analysis not described
Zhou et al., 2016 [20]	Neutral	√	x	√	√	x	√	√	x	x	√	Prospective participants were excluded if they were unable to use a smartphone. Statistical analysis was unclear

√ = response of “yes” to the validity question; x = response of “no” to the validity question; ^a^ Assessed using The Quality Criteria Checklist for Primary Research [14]; ^b^ Validity items: [1] research question stated; [2] subject selection free from bias; [3] comparable study groups; [4] method for withdrawals described; [5] blinding used; [6] interventions described; [7] outcomes stated, measurements valid and reliable; [8] appropriate statistical analysis; [9] appropriate conclusions, limitations described; [10] funding and sponsorship free from bias. Validity items 2, 3, 6, 7 must be satisfied for a positive quality rating.

## Appendix A

**Table A1:** Secondary outcomes of studies exploring the effect of using mobile devices to record food or nutrition intake on diabetes control and nutrition outcomes.

Author	Fasting BGL	BMI, Weight and Anthropometry	Lipids	Food Intake	Satisfaction and Useability	Uptake and Engagement
Drion et al., 2015 [15]	-	-	-	-	DBEES app rated 77 using the System Usability Scale (>70 is acceptable). No specific evaluation of the dietary component.	-
Forjouh et al., 2014 [17]	-	“modest reductions” in BMI in all groups. data not provided.	-	PDA group ate more high fat foods (*p <* 0.004). Data not reported.	-	Interaction with dietary record component specifically not reported. CDSMP + PDA group—verage of 359 entries/year. PDA group—average of 342 entries/year.
Quinn et al., 2011 [18]	-	-	No change in TAG, LDL or HDL within groups, with no difference between groups (*p* not reported). Significant reduction in total cholesterol within the coach group but no change within other groups and no difference in change between groups (*p* not reported).	-	-	-
Rossi et al., 2013 [16]	Significant reduction within control group, no change within intervention group. No significant difference in change between groups (*p =* 0.07).	No change in weight within intervention or control group. No difference in change in weight between groups (*p =* 0.85).	No change within groups and no significant difference in change between groups for total cholesterol (*p =* 0.47), HDL (*p =* 0.71) or TAG (*p =* 0.22). Significant reduction within intervention group but not control group, with no difference in change between groups for LDL (*p =* 0.61).	-	-	-
Waki et al., 2014 [19]	Significant difference in change between groups (*p =* 0.019) favouring the intervention.	No difference in change in BMI between groups (*p =* 0.062).	No difference in change in HDL (*p =* 0.36), LDL (*p =* 0.43) or total cholesterol (*p =* 0.24) between groups.	-	Most patients responded favourably to satisfaction questions.	Average time spent using the system was 22.5 min/day (relates to whole app). On average 40% recorded dietary data and 69% photographed the meal. Recording of dietary data declined from 54% to 27% of patients between the first 2 weeks and the last 2 weeks. This was also observed for photos of meals—77% first 2 weeks, 51% last 2 weeks.
Zhou et al., 2016 [20]	Significant reduction within both groups, with significant difference in change between groups in favour of the intervention (*p <* 0.01).	No change in weight, BMI or waist circumference within either group, and no difference in change between groups (*p* not reported).	No change in LDL within either group and no difference in change between groups (*p* not reported).	-	84% of patients in the intervention group were satisfied with the app.	
Tsang 2001 [23]	-	-	-	-	95% reported the system was easy to use. 63% reported it was useful in evaluating eating habits. 36% experienced technical problems.	Variation in the frequency of data transmission and analysis: 15% ≥7/week, 11% 5–6/week, 21% 3–4/week, 37% 1–2/week, 15% <1/week. The majority (73%) of participants transmitted data for analysis for 3 meals per occasion.
Rossi et al., 2010 [22]	No change within either group, and no difference in change between groups (*p =* 0.13).	Significant increase in weight within the control group but no change within the intervention group. No difference in change in weight between groups (*p =* 0.22).	No change within either group for TAG, but significant difference in change between groups in favour of the intervention (*p =* 0.04). No change within either group and no difference in change between groups for total cholesterol (*p =* 0.33) or LDL (*p =* 0.79). Significant increase in HDL within control group but no change within intervention group and no difference in change between groups (*p =* 0.14).	-	-	Interaction with dietary record component specifically not reported. The median (range) number of text messages sent by each patient during the study was 52 (6–75), whereas the number of text messages sent by the clinician was 39 (22–70).
Holman et al., 2014 [21]	-	No change in weight within any groups. No difference in change in weight between groups (*p* not reported).	-	No difference between groups in change in intake of fruits, vegetables, meat, chocolate and fish (*p* not reported).	-	Interaction with dietary record component specifically not reported. 39% high users in FTA group and 34% in FTA HC group (where high user ≥5 BGL measurements and ≥ 50 interactions with the diary). Those aged ≥63 years used the app significantly more than younger participants (*p =* 0.045).

BGL, blood glucose level; BMI, body mass index; SD, standard deviation; HDL, high density lipoprotein; LDL, low density lipoprotein; TAG, triglyceride; app, application; CDSMP, chronic disease self-management program; PDA, personal digital assistant; FTA, Few Touch Application; HC, health counseling; DBEES, Diabetes Under Control.
